# Geographic divergence of methicillin-resistant *Staphylococcus aureus* ST5-SCC*mec*I in the aftermath of a major earthquake and tsunami: impact of a plasmid harboring heavy metal resistance genes

**DOI:** 10.1128/mbio.03669-24

**Published:** 2025-03-19

**Authors:** Jose R. W. Martínez, Manuel Alcalde-Rico, Estefanía Jara-Videla, Jinnethe Reyes, Lina P. Carvajal, Sandra Rincon, Rafael Ríos, Lorena Diaz, Ana Quesille-Villalobos, Roberto Riquelme-Neira, Lina Rivas, Ahmed M. Moustafa, Blake Hanson, Eduardo A. Undurraga, Jorge Olivares-Pacheco, Patricia García, Rafael Araos, Paul J. Planet, César A. Arias, Jose M. Munita

**Affiliations:** 1 Genomics & Resistant Microbes Group (GeRM), Instituto de Ciencias e Innovación en Medicina (ICIM), Facultad de Medicina Clinica Alemana, Universidad del Desarrollo, Santiago, Chile; 2 Multidisciplinary Initiative for Collaborative Research On Bacterial Resistance (MICROB-R), Santiago, Chile; 3 Grupo de Resistencia a los Antibióticos en Bacterias Patógenas y Ambientales (GRABPA), Instituto de Biología, Pontificia Universidad Católica de Valparaíso, Valparaiso, Chile; 4 Instituto de Biomedicina de Sevilla (IBiS), Hospital Universitario Virgen Macarena, CSIC, Universidad de Sevilla, Sevilla, Spain; 5 Molecular Genetics and Antimicrobial Resistance Unit, Universidad El Bosque, Bogota, Colombia; 6 Núcleo de Investigaciones Aplicadas en Ciencias Veterinarias y Agronómicas, Facultad de Medicina Veterinaria y Agronomía, Universidad de las Américas, Santiago, Chile; 7 Division of Pediatric Infectious Diseases, Children’s Hospital of Philadelphia, Philadelphia, Pennsylvania, USA; 8 Department of Pediatrics, Perelman College of Medicine, University of Pennsylvania, Philadelphia, Pennsylvania, USA; 9 Division of Gastroenterology, Hepatology, and Nutrition, Children’s Hospital of Philadelphia, Philadelphia, Pennsylvania, USA; 10 Center for Infectious Diseases, School of Public Health, University of Texas Health Science Center, Houston, Texas, USA; 11 Escuela de Gobierno, Pontificia Universidad Católica de Chile 28033 , Santiago, Chile; 12 Research Center for Integrated Disaster Risk Management (CIGIDEN), Santiago, Chile; 13 CIFAR Azrieli Global Scholars Program, CIFAR 145760 , Toronto, Canada; 14 Departamento de Enfermedades Infecciosas, Escuela de Medicina, Pontificia Universidad Católica de Chile, Santiago, Chile; 15 American Museum of Natural History, New York, New York, USA; 16 Division of Infectious Diseases, Houston Methodist Hospital, Houston, Texas, USA; 17 Center for Infectious Diseases, Houston Methodist Research Institute, Houston, Texas, USA; 18 Department of Medicine, Weill Cornell Medical College, New York, New York, USA; University of California, Irvine, Irvine, California, USA

**Keywords:** MRSA, ST5-SCC*mec*I, geographic divergence, earthquake, tsunami, heavy metals

## Abstract

Methicillin-resistant *Staphylococcus aureus* (MRSA) is a major public health menace. The global spread of MRSA is characterized by successive waves of epidemic clones dominating specific geographical regions. The acquisition of genes encoding resistance to heavy metals (HMRGs) is thought to be a key feature in the geographic divergence of MRSA. However, the cause-effect relationship between the presence of HMRGs and the divergence of MRSA clones remains to be clarified. In this study, we assessed the role that HMRGs may have played in the evolutionary divergence of the MRSA ST5-SCC*mec*I lineage in Latin America. We conducted a genomic characterization of 113 MRSA clinical isolates from six Latin American healthcare centers, including 53 isolates collected from two cities in Chile (Santiago and Concepción). We found a plasmid (pSCL4752) harboring arsenic, cadmium, and mercury resistance genes in 65% (*n* = 71) of the ST5-SCC*mec*I isolates. We also observed a geographic divergence associated with the presence of pSCL4752 in Chilean isolates, with a higher frequency in isolates from Concepción (88%) compared to Santiago (29%). Interestingly, a molecular clock analysis revealed that this divergence occurred in the aftermath of an 8.8 Mw earthquake and tsunami that struck the Concepción area in 2010. Moreover, our results demonstrate that the carriage of pSCL4752 can be beneficial or detrimental for ST5-SCC*mec*I isolates, depending on the environmental availability of these heavy metals. Our results suggest that the divergence of the ST5-SCC*mec*I MRSA lineage in Latin America could have been fostered by environmental disasters and influenced by the presence/absence of HMRGs harbored in a plasmid.

**IMPORTANCE:**

Methicillin-resistant *Staphylococcus aureus* (MRSA) is a major cause of life-threatening infections worldwide and a growing public health concern. The rise of antibiotic-resistant bacteria, such as MRSA, is often linked to genetic adaptations that enhance their survival. Our research sheds light on how environmental changes, such as those triggered by a natural disaster, can influence the evolution and geographic spread of a highly resistant MRSA lineage in Latin America. We identified a plasmid carrying genes for resistance to arsenic, cadmium, and mercury, which was associated with the geographic divergence of the ST5-SCC*mec*I MRSA lineage, with striking differences in its prevalence between regions affected by a major earthquake and tsunami. By linking environmental events to pathogen evolution, our study highlights the role of ecological pressures in the spread of MRSA. These findings emphasize the need to integrate environmental monitoring into public health strategies to better understand the global challenge of antimicrobial resistance.

## INTRODUCTION

Methicillin-resistant *Staphylococcus aureus* (MRSA) infections are a major public health problem and a top priority (1, 2). MRSA spread has distinctive epidemiologic patterns with genetic lineages restricted to specific geographical areas (3, 4). The widespread dissemination of MRSA over time is often driven by “waves” of clonal replacements, where novel lineages replace predominant regional clones (5). While the underlying factors driving clonal MRSA replacement events remain unclear, this phenomenon has been widely reported in different parts of the world, including Latin America (6–9).

The acquisition of genetic traits conferring resistance to heavy metals seems to be a key feature in the divergence and spread of successful MRSA lineages. Indeed, one of the main features in the genomic evolution of a major community-acquired MRSA (CA-MRSA) lineage, the USA300 clone, was the parallel acquisition of two horizontally acquired genetic elements: the arginine catabolic mobile element (ACME) in the North American USA300 clade (USA300-NAE), and the copper (Cu) and mercury (Hg) resistance mobile element (COMER) in South American clade (USA300-SAE) (10). Of note, apart from the arginine metabolism machinery, USA300-NAE ACME also harbored *copX*(B), a copper (Cu) resistance gene also observed in other successful MRSA clones (11). Other studies have also suggested a possible evolutionary advantage of acquiring heavy metal resistance genes (HMRGs) in the emergence of new MRSA lineages, further supporting the hypothesis that mobile genetic elements harboring HMRGs might play a role in the successful dissemination of MRSA (12, 13).

One of the most successful MRSA clones in Latin America has been the Chilean-Cordobes (ChC) clone, a healthcare-associated ST5-SCC*mec*I lineage first described in 1998 in Chile and Argentina (14, 15). While the ChC clone was almost completely replaced in Colombia and Ecuador during the 2000s by the above-mentioned CA-MRSA USA300-SAE clone (3, 16), it remained largely dominant in the South Pacific coast of Latin America, including countries like Chile and Peru (17–19). In a study analyzing clinical MRSA isolates collected between 2000 and 2016 in Chile, we recently reported that the ChC clone remained the most frequent MRSA lineage in Chile. However, a gradual reduction in its frequency has been observed over time (20). Of note, we also found that ~80% of the isolates belonging to the ST5-SCC*mec*I lineage harbored HMRG which included arsenic (As), cadmium (Cd), and Hg resistance genes (20). However, the role of these HMRGs in the dissemination and evolution of the ChC clone remains unclear.

To explore the role of HMRGs in the evolution of the ST5-SCC*mec*I lineage, we performed a detailed genomic characterization of 113 MRSA isolates recovered from bloodstream infections in six Latin American countries (3), all of which had been previously characterized as part of the ChC clone by pulse-field gel electrophoresis (PFGE). We explored the genomic context of HMRGs identifying a plasmid carrying As, Cd, and Hg resistance genes. We then dated the major divergence events associated with the presence of this plasmid in Chile to assess their impact on the evolution of the MRSA ST5-SCC*mec*I lineage and carried out phenotypic assays to evaluate the activity and fitness cost of these genes.

## RESULTS

### Closely related ST5-SCC*mec*I MRSA genomes presented a similar geographic distribution

**Fig 1:**
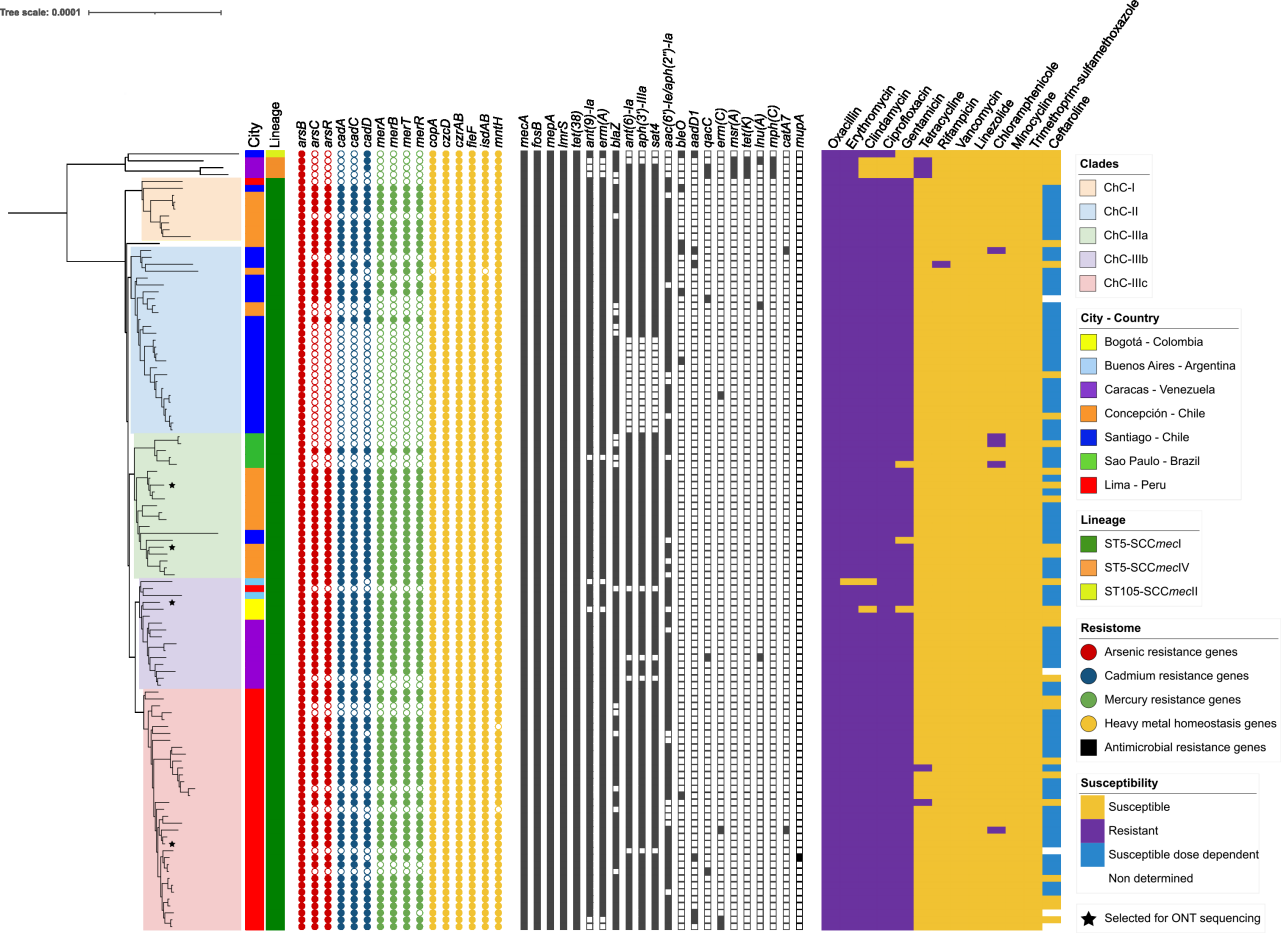
Core genome-based phylogenomic reconstruction of the 113 Latin American ChC clone MRSA genomes. The phylogenomic reconstruction was rooted at the midpoint of genomic distances. The most important clades are represented by colors within the reconstruction. The inner colored bar indicates the city of origin of each isolate. The second colored bar indicates the MRSA lineage defined by the combination of ST and SCC*mec* type. The heavy metal resistance genes are represented by colored circles indicating presence (filled circles) or absence (empty circles). Antibiotic resistance genes are depicted by black squares indicating presence (filled squares) or absence (empty squares). Categorical susceptibility to antimicrobials was determined by CLSI 2024 guidelines and is represented by purple rectangles for resistant (including intermediate category), dark yellow rectangles for susceptible, and light blue rectangles for susceptible dose-dependent (only for ceftaroline). Isolates selected for long-read sequencing are indicated by black stars.

Our study included 113 MRSA isolates identified as the ChC clone by PFGE and recovered from bacteremia in seven Latin American hospitals: Lima, Peru (*n* = 37, 32.7%); Santiago, Chile (*n* = 28, 24.8%); Concepción, Chile (*n* = 25, 22.1%); Caracas, Venezuela (*n* = 13, 11.5%); Sao Paulo, Brazil (*n* = 5, 4.4%); Bogotá, Colombia (*n* = 3, 2.7%); and Buenos Aires, Argentina (*n* = 2, 1.8%). As typically described for the ChC MRSA clone, isolates exhibited resistance to ciprofloxacin, gentamicin, erythromycin, and clindamycin, along with susceptibility to tetracyclines, cotrimoxazole, rifampicin, vancomycin, and linezolid (Fig. 1; Table S1). Consistent with a previous report, only 27% of the isolates were susceptible to ceftaroline, while the remaining 73% exhibited minimum inhibitory concentrations (MICs) in the susceptible dose-dependent range, as per the Clinical and Laboratory Standards Institute (CLSI) 2024 breakpoints (Fig. 1; Table S1) (21). No differences in the antimicrobial susceptibility pattern were observed across the seven healthcare centers (Fig. 1; Table S1). Our genomic analyses classified all 113 isolates as members of clonal complex 5, with 109 (96%) of them being ST5-SCC*mec*I, 3 (3%) being ST5-SCC*mec*IV, and 1 (1%) being ST105 SCC*mec*II.

To further explore the genomic relatedness of our collection of invasive ChC MRSA isolates, we performed a core genome-based phylogeny (Fig. 1). Our phylogenomic reconstruction showed that the 109 ST5-SCC*mec*I genomes (96%) were grouped into three well-defined clades (ChC-I, II, and III) that followed a geographic pattern. Clades ChC-I (*n* = 10) and ChC-II (*n* = 27) predominantly consisted of MRSA genomes from Chile (35/37). Clade ChC-III gathered the largest number of genomes (*n* = 72) and was further split into three sub-clades: (i) ChC-IIIa (*n* = 21) included 16 isolates from Chile and all five genomes from Brazil; (ii) ChC-IIIb (*n* = 16) grouped all isolates from Venezuela, Colombia, and Argentina; and (iii) ChC-IIIc (*n* = 35) only included isolates recovered from Peru. Interestingly, a geographically divergent pattern was also observed between both cities within Chile, with clade ChC-II mainly composed of genomes from isolates recovered in Santiago (central Chile), and clades ChC-I and ChC-IIIa mainly composed of MRSA genomes obtained from Concepción (southern Chile).

### High frequency of HMRGs in the Latin American ChC ST5-SCC*mec*I MRSA clone

To further characterize the genomes of ST5-SCC*mec*I MRSA circulating in Latin America and to explore the phylogeographical clustering observed with the core-genome phylogeny, we conducted a comprehensive resistome analysis, focusing on the presence of HMRGs and other antimicrobial resistance determinants (Fig. 1). Our analysis showed that >90% of the genomes contained heavy metal resistance genes including *arsB* (As resistance), *czcD* and *czrAB* (Cd and zinc resistance), and *copA* (Cu exporting). In addition, key antimicrobial resistance genes were found in high frequency, such as *mecA* (resistance to penicillins and cephalosporins), *fosB* (fosfomycin resistance), *mepA* (fluoroquinolone resistance), *lmrS* and *ermA* (macrolides and lincosamides resistance), *aph(9)-Ia* (aminoglycosides resistance), and *tet38* (low-level tetracyclines resistance). Other frequently encountered antimicrobial resistance genes present in 80%–90% of the genomes included *blaZ* (penicillins), *aph(3′)-IIIa*, *ant(6)-Ia*, and *aac(6′)-Ie/aph(2″)-Ia* (aminoglycosides) (Table S2). Additionally, the heavy metal resistance operons *arsCR* (As resistance), *cadACD* (Cd resistance), and *merABTR* (Hg resistance) were identified in 60%–70% of the genomes (Fig. 1). Interestingly, 71 (63%) genomes co-harbored all resistance determinants for resistance to As (*arsBCR*), Cd (*cadACD*), and Hg (*merABTR*). Further analysis of these HMRGs considering the above-described ST5-SCC*mec*I sub-clades revealed a high prevalence of As, Cd, and Hg resistance genes in clades ChC-I (78%) and ChC-III (84%). In contrast, only 8 out of 27 (30%) genomes from clade ChC-II carried any of the aforementioned HMRGs (Fig. 1). These findings suggested an association between specific sub-clades of the ST5-SCC*mec*I MRSA lineage and the presence of HMRGs, mainly observed in Chilean genomes from Santiago (clade ChC-II) and Concepción (clades ChC-I and ChC-IIIa) with low and high frequency of HMRGs, respectively.

### The dissemination of HMRGs was driven by a conserved plasmid (pSCL4752)

**Fig 2:**
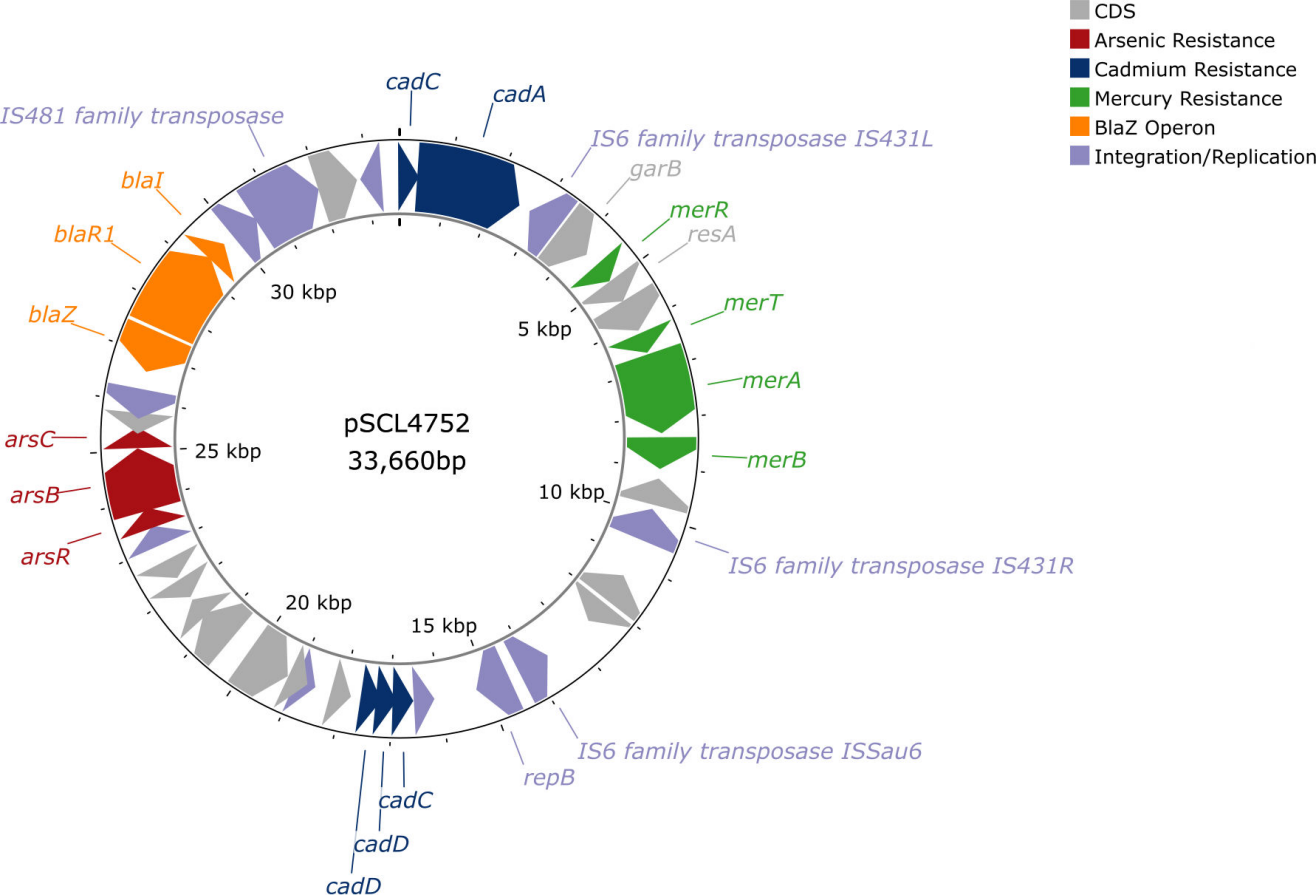
Schematic representation of plasmid pSCL4752. The circularized pSCL4752 plasmid includes the annotations of the coding sequences (gray arrows), while genes conferring resistance to heavy metals are color-coded: arsenic (red arrows), cadmium (blue arrows), and mercury (green arrows). The *blaZ* operon is shown in orange arrows. Transposases and replicases are marked in purple arrows.

To determine the genetic context of these HMRGs, we performed hybrid assemblies using short-read sequencing and long-read sequencing (LRS) of four representative strains (one from each of the cities in Chile [Santiago and Concepción], one from Colombia, and one from Peru) harboring As, Cd, and Hg resistance determinants (Fig. 1). All assemblies generated a complete genome of ~3,050,000 bp with a GC content of 32.9%, composed of two circularized contigs, including the chromosome (~3,000,000 bp), and a plasmid of ~37,000 bp (Table S3). This plasmid shared extensive identity (99%) with pCM05, a plasmid previously identified in a linezolid-resistant ST5-SCC*mec*I MRSA strain isolated in Medellin, Colombia (NC_013323.1) (22). Our plasmid, designated pSCL4752, encoded a total of 41 CDSs, including all horizontally acquired HMRGs previously mentioned (*arsBCR*, *cadA*, *cadC* [x2], *cadD* [x2], and *merABTR*), and a copy of the *blaIRZ* operon, which encodes the staphylococcal penicillinase BlaZ (Fig. 2). pSCL4752 also contained two duplicated invertases (*bin3* and *hin*), five transposases (IS*431L*, IS*431R*, IS*Sau6*, IS*Bli29*, and IS481), and a plasmid replication initiator protein (*repB*), along with three replication proteins and several hypothetical proteins (Fig. 2). *merABTR* and the reductase genes *resA* and *garB* were flanked by two IS26 family transposases (*IS431L* and *IS431R*), suggesting the presence of a mobile Hg resistance transposon. To determine the presence of the pSCL4752 plasmid within our collection, all genome assemblies were analyzed searching for contigs with high nucleotide sequence identity that covered the full plasmid length. Our analysis predicted the presence of pSCL4752 in 72 out of 109 (66%) of the ST5-SCC*mec*I genomes. No significant differences in the phenotypic antimicrobial susceptibility pattern were observed between isolates harboring and those not harboring pSCL4752 (*P* ≥ 0.05). Notably, a significant difference in plasmid carriage was observed among Chilean isolates: 88% of isolates from Concepción (clades CHC-I and ChC-IIIa) harbored the plasmid, compared to only 29% of those from Santiago (clade ChC-II) (*P* < 0.05), suggesting that local geographical selective pressures might have played a role in the acquisition or retention of pSCL4752.

### Geographical divergence of the ST5-SCC*mec*I MRSA lineage in Chile was associated with the presence of pSCL4752

**Fig 3:**
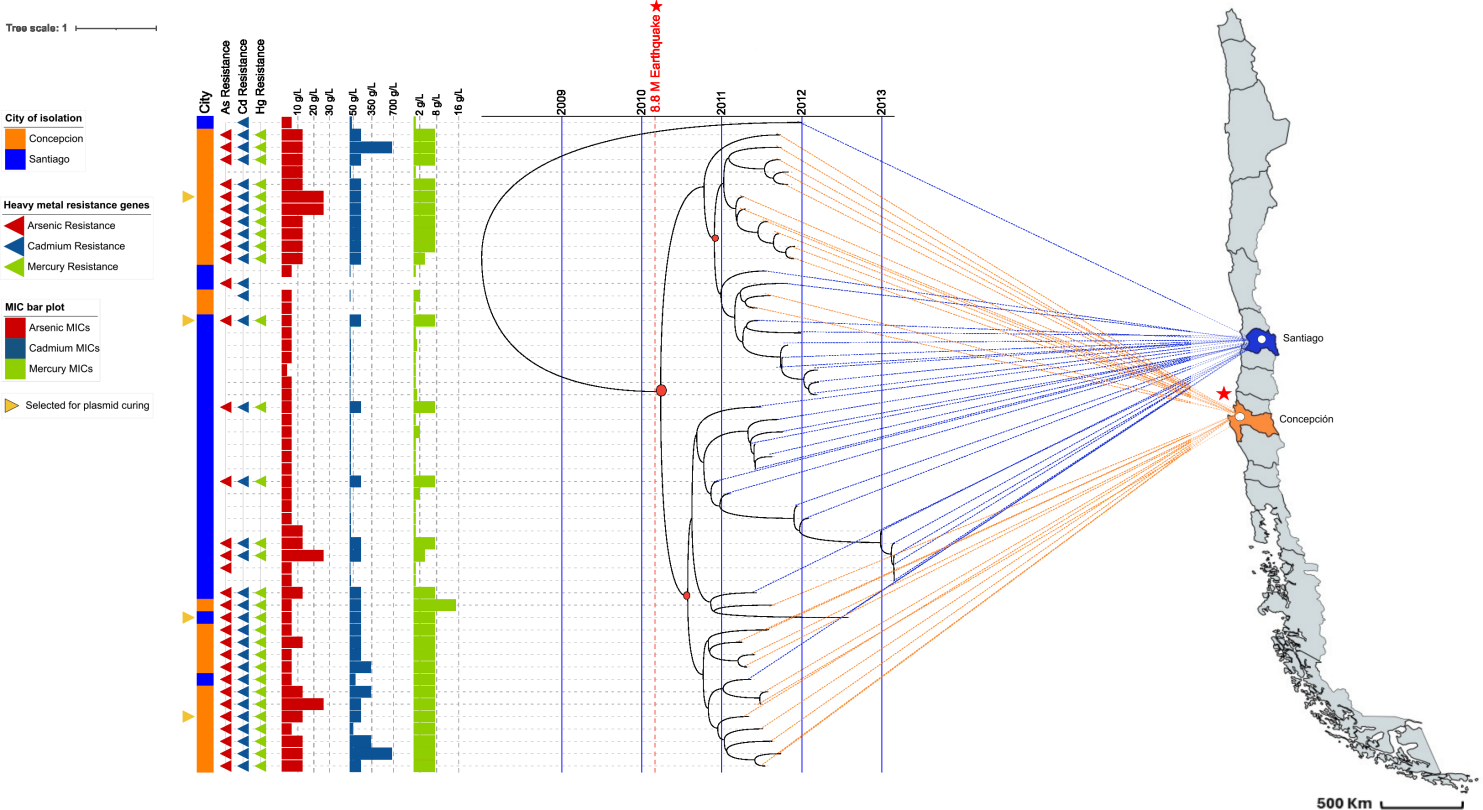
Bayesian molecular clock analysis of the Chilean ST5-SCC*mec*I genomes. The tips of each branch of the tree correspond to the isolation date, and the time scale is displayed at the top of the tree. The red-colored circles in the tree represent the main divergence events. The colored band shows the city of origin. The triangles show the presence of the heavy metal resistance genes: red, arsenic resistance genes (*arsB* and/or *arsC*); blue, cadmium (*cadA*, *cadC*, and/or *cadD*); and green, mercury (*merA*, *merB*, *merT*, and/or *merR*). The bar plot shows the minimum inhibitory concentration of arsenic (red), cadmium (blue), and mercury (green) for each strain tested. Isolates selected for plasmid curing experiments are indicated in yellow triangles. Each tip is connected to the city of origin of the genome on the map by dashed lines. The star represents the epicenter of the earthquake and tsunami.

To shed light on the evolutionary dynamics related to the geographic divergence of Chilean MRSA isolates harboring pSCL4752, we performed a Bayesian molecular clock analysis including all ST5-SCC*mec*I genomes recovered from Santiago and Concepción (Chile). Our analysis dated the most recent common ancestor (MRCA) of the collection to 2008 (95% high posterior density interval 2007.03–2008.77) (Fig. 3). The molecular clock revealed a major divergence event in March 2010, which was quickly followed by two parallel divergence events between September and November of 2010. As shown in Fig. 3, both of these events grouped isolates into four clades highly related to the origin city (Santiago or Concepción) and to the presence/absence of pSCL4752 (*P* < 0.05). Of note, the divergence events were dated just months after a major earthquake and tsunami (8.8 Mw) struck the Concepción area on 27 February 2010. These results further suggested that the geographical difference in pSCL4752 carrying might be related to local selective factors and not driven by the natural evolution of MRSA genomes.

### Isolates harboring the pSCL4752 plasmid exhibited increased resistance to heavy metals

**Fig 4:**
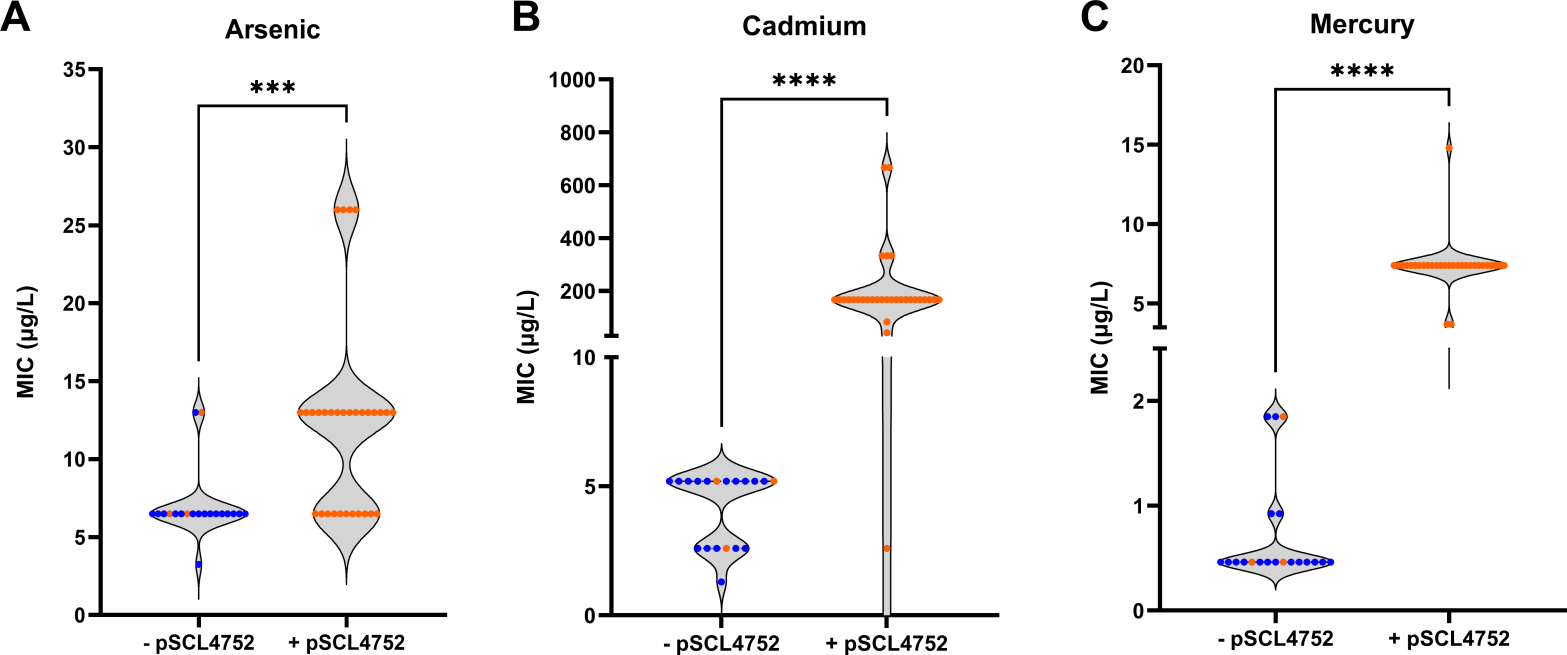
Phenotypical effect of the presence of the pSCL4752 plasmid in Chilean clinical isolates. Broth microdilution MICs of the 53 Chilean clinical isolates to arsenic (**A**), cadmium (**B**), and mercury (**C**). The MIC value was determined as the minimal concentration that inhibits bacterial growth. Statistical analysis was performed with the non-parametric Mann-Whitney test. *P < 0.05, ***P < 0.001, ****P < 0.0001, ns = non-significant.

**Fig 5:**
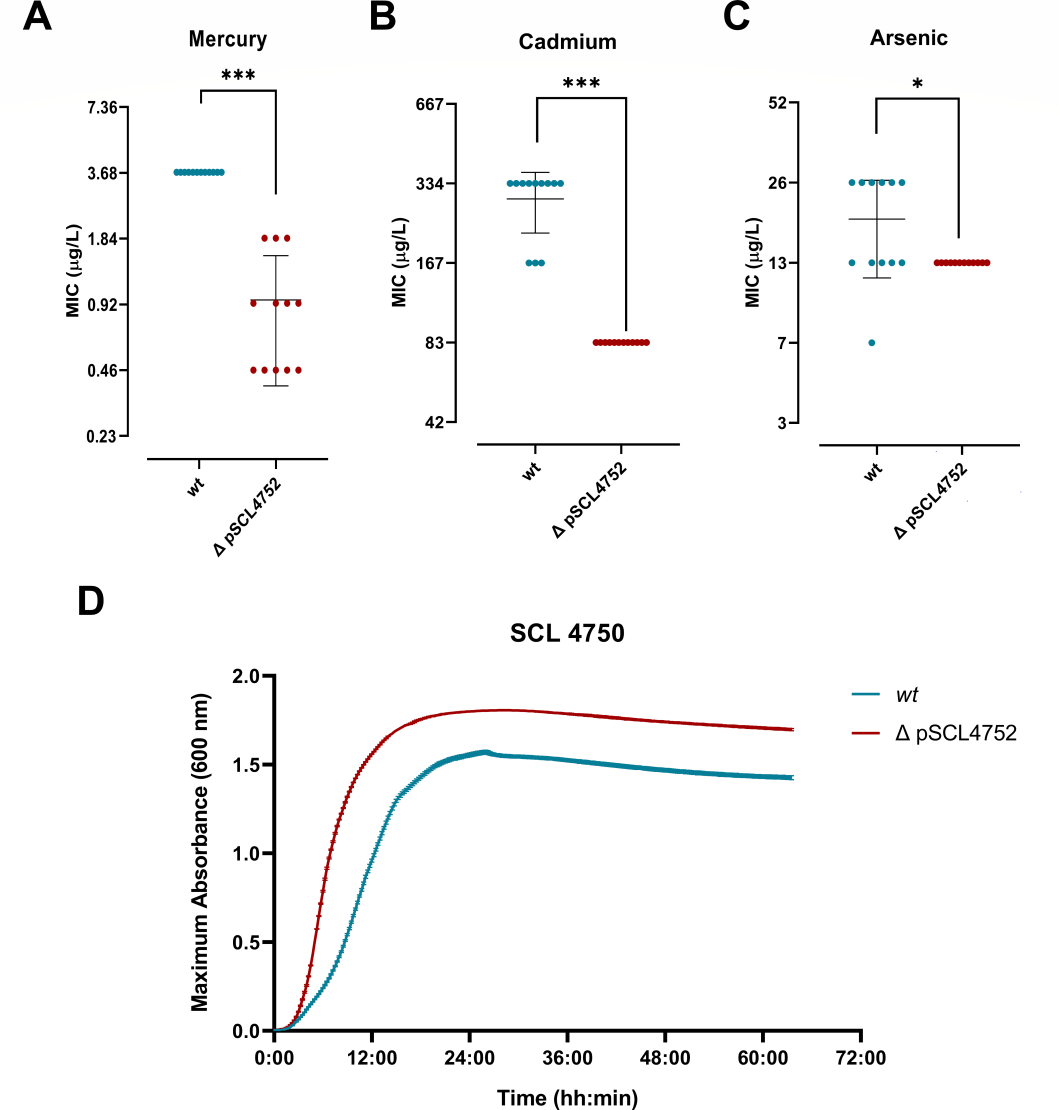
Phenotypical analysis of four pSCL4752-cured strains. MIC determination by broth microdilution method to mercury (**A**), cadmium (**B**), and arsenic (**C**) in four MRSA isogenic clone strains carrying the plasmid (*wt*) and plasmid-cured (ΔpSCL4752). The MIC value was determined as the minimal concentration that inhibits bacterial growth. (**D**) Growth curve of representative plasmid-cured strain (SCL4750). The color of the curves represents the plasmid curing treatment, being dark red for treated and green for non-treated. The *X*-axis represents the time, and the *Y*-axis represents the OD_600_. All the curves were performed in triplicates from at least two independent experiments, and the figure shows means ± standard deviations. Statistical analysis was performed with the non-parametric Wilcoxon matched-pairs signed rank test. *P < 0.05, ***P < 0.001, ****P < 0.0001, ns = non-significant.

To assess the functionality of the HMRGs contained in pSCL4752, we determined the minimal inhibitory concentrations to As, Cd, and Hg in isolates from Santiago and Concepción (Fig. 4). Plasmid-harboring isolates exhibited significantly higher MICs to As, Cd, and Hg than their pSCL4752 negative counterparts (*P* < 0.0001), with the following MIC_50/90_ values: 13/26 mg/L vs 6.5/13 mg/L for As; 167/334 mg/L vs and 5.2/5.2 mg/L for Cd; and 7.4/7.4 mg/L vs 0.4/1.9 mg/L for Hg (Fig. 4). To further corroborate the activity of the HMRGs contained in pSCL4752 and to evaluate the fitness cost associated with its carriage, pSCL4752 was cured from four representative strains through successive passages in the absence of heavy metals. pSCL4752-cured strains exhibited significantly lower MICs (*P* < 0.05) to Hg, Cd, and As, as compared to their isogenic ancestral counterparts (Fig. 5A through C). In addition, growth curves performed in media not supplemented with heavy metals demonstrated plasmid-cured strains grew significantly faster (doubling time 42 min ± 5.7 vs 59 min ± 3.8, respectively, *P* < 0.05) and reached a higher optical density at 600 nanometers (OD_600_ )(1.761 ± 0.028 vs 1.420 ± 0.390, *P* < 0.0001) than their isogenic parental strains harboring pSCL4752 (Fig. 5, lower panel). These results suggested that while pSCL4752 introduces a fitness cost in the absence of selective pressure, its carriage may confer an evolutionary advantage to ST5-SCC*mec*I isolates in the presence of heavy metals.

## DISCUSSION

A robust body of evidence has shown an association between the acquisition of HMRGs and the emergence of resistant pathogens (10, 23, 24). However, the role of plasmids harboring HMRGs in the evolution and dissemination of MRSA lineages remains largely unexplored. In this study, we provide a detailed genomic analysis of the ST5-SCC*mec*I lineage, a successful MRSA clone disseminated across different countries of Latin America. Our findings suggest that this lineage exhibits significant genomic and phylogeographic divergence that is associated with the presence of pSCL4752, a plasmid harboring key heavy metal resistance determinants.

One of our most interesting findings was the geographical divergence among the Chilean ST5-SCC*mec*I MRSA isolates. Such divergence was highly associated with a higher frequency of isolates carrying pSCL4752 in Concepción as compared to Santiago and suggested that local selective pressures likely shape within-lineage MRSA evolution. Environmental contamination with heavy metals has been associated with horizontal gene transfer and with the selection of non-pathogenic organisms harboring plasmids containing heavy metal resistance traits (23, 25). Although the environmental contamination with heavy metals in Chile has been poorly explored, historical records before the 2010 earthquake reported similar levels of environmental heavy metals in Concepción and Santiago, reducing the potential role of prolonged heavy metal exposure as a main driver of the geographic MRSA divergence (26, 27). However, several studies have shown that tsunamis and other major catastrophic events may release and mobilize heavy metals from marine sediments (28–31). This could be important for two reasons: (i) previous evidence suggests that the Concepción Bay marine sediment has high levels of Hg and other heavy metals (26, 27), and (ii) on 27 February 2010, a massive earthquake (8.8 Mw) and a subsequent tsunami significantly impacted Concepción Bay (32). Interestingly, our molecular clock analyses estimated that the initial divergence event leading to the selection of pSCL4752-harboring isolates in Concepción occurred in March 2010. Although there have not been any measurements of environmental heavy metals after the 2010 earthquake, a previous study did observe a significant increase in heavy metals in mollusks—usually used as biosensors of heavy metal contamination—collected off the coast of Concepción after the tsunami that followed the earthquake (33–35). These data suggest that the 2010 earthquake and tsunami may have released heavy metals into the environment, likely acting as a selective pressure driving the divergence observed in the ST5-SCC*mec*I MRSA clone in Chile. Indeed, our phenotypic experiments suggest that while pSCL4752 might provide an adaptive advantage in the presence of heavy metals, it has a fitness cost in the absence of selective pressure, reinforcing the potential role of local selective pressures on the evolutionary divergence of this MRSA clone.

Our phenotypic analyses revealed an increase in the MICs to Hg and Cd of at least fourfold in isolates carrying the pSCL4752, suggesting that these heavy metals could be the most probable selective pressure for the plasmid maintenance. Historical records of Hg accumulation in Concepción Bay further support this hypothesis, since Hg is highly volatile at room temperature and due to its geochemical properties, can be transported over long distances as particulate, dissolved, or gaseous form before being deposited in soil or surface water (26, 36–38). Although direct evidence of Hg exposure in Concepción’s human population after the earthquake is unavailable, it is well-established that the release of heavy metals following seismic events can adversely impact human health both directly (acute toxicity) and indirectly, with long-term consequences such as latent or persistent infections (39). The movement of marine sediments caused by tsunamis significantly increases heavy metal concentration in topsoil, irrigation water, drinking water, and rivers, leading to widespread contamination (40, 41). Indeed, suburban gardens and agricultural lands have shown increased heavy metal concentrations after earthquakes, resulting in potential contamination of food crops grown on these soils (42, 43). Taken together, this evidence suggests that human exposure to heavy metals through environmental contamination after the earthquake may have played a role in selecting MRSA isolates harboring the pSCL4752 plasmid.

We find a high frequency of the plasmid pSCL4752 in ST5-SCC*mec*I isolates from all the countries included in the study, except for Brazil, where only one out of five isolates carried the plasmid. This observation underscores the evolutionary success of the pSCL4752 plasmid and suggests that heavy metal resistance traits provide a significant adaptive advantage in the emergence and maintenance of MRSA lineages. Consistent with our findings, previous studies have shown that some MRSA clones have maintained mobile genetic elements containing HMRGs despite a fitness cost, due to adaptive advantage beyond heavy metal resistance (12, 13). Indeed, a horizontally transferred copper-resistance locus provided increased survival in macrophages and was associated with the co-carriage of crucial antimicrobial resistance determinants in USA300 (13, 44). In our case, we detected a *blaIRZ* operon in pSCL4752, which may also be related to the selection of the plasmid. However, we found a significantly high proportion of isolates lacking pSCL4752, and hence lacking HMRGs, but containing the *blaIRZ* operon. The results suggest that the plasmid was most likely selected by heavy metals and not by a potential advantage provided by this antimicrobial resistance operon. Additionally, we find by our genomic analyses that Hg resistance genes could be transposable, as they are contained within a transposon-like structure flanked by two IS26 family transposases (IS431L and IS431R). This element has also been found in the chromosome linked to the SCC*mec* element in other MRSA lineages, including the COMER element of the USA300 Latin American variant (10). This phenomenon further suggests that mobile heavy metal resistance determinants might play a major role in the selection of successful MRSA lineages.

Our core genome-based phylogeographic analyses of ST5-SCC*mec*I lineage showed a substantial genomic heterogeneity strongly associated with the city of origin. These results align with previous data suggesting a higher geographical diversity in MRSA isolates belonging to clonal complex 5 (which includes ST5-SCC*mec*I) as compared to other MRSA lineages (4). Geographic genomic heterogeneity has also been observed in other MRSA lineages such as ST105 and ST239, both of which underwent marked divergence within different regions of Brazil (9, 45). The divergence events that generated the North and South American USA300 clones subsequently led to further rapid clonal expansion across different geographic regions (16). Furthermore, the appearance of two predominant variants of the USA300 clone in an outbreak in New York suggested that MRSA clones may undergo genomic divergences even within the same geographical area and genetic lineage (46). These observations highlight the complex evolutionary dynamics of MRSA and the significant role that local environmental factors play in shaping the genomic landscape of these pathogens.

In conclusion, we used genomic data from clinical isolates of the ST5-SCC*mec*I ChC MRSA clone to describe a major evolutionary divergence event associated with the acquisition of a plasmid harboring heavy metal resistance genes. We observed that the divergence follows a spatiotemporal pattern coinciding with an extreme natural event, the 2010 earthquake and tsunami, probably due to the heavy metal mobilization that has been previously associated with such a natural disaster (29, 31, 47). Indeed, we found experimental evidence of a possible link between the presence of HMRGs-containing pSCL4752 plasmid and the fitness cost associated, which may be interpreted as either an evolutionary advantage or disadvantage, depending on the presence or absence of heavy metals in the environment, respectively. Unfortunately, there is no record of heavy metal concentrations in Santiago or Concepción in the aftermath of the 2010 earthquake, limiting the comparison between the minimum inhibitory concentrations obtained in this study and real-world conditions. Moreover, Chile was the only country where isolates were collected from two different cities. Therefore, we cannot discard the possibility that similar divergence events associated with the loss of pSCL4752 could have occurred in other regions of Latin America that were not affected by the earthquake of 2010. Improving our understanding of how chronic exposure and adaptation to environmental pollution associated with extreme events could affect the emergence of antimicrobial resistance determinants is critical to avoid a potential future health crisis. Our results highlight the urgent need for additional research on environmental risk factors and climate change associated with the emergence of MRSA and other antimicrobial-resistant organisms.

## MATERIALS AND METHODS

### Strain collection

We studied a collection of 113 MRSA isolates recovered from six Latin American countries (Argentina, Brazil, Chile, Colombia, Peru, and Venezuela) identified as ChC by PFGE in a previously published prospective cohort study (3). Isolates were recovered from adult patients diagnosed with *S. aureus* bacteremia while admitted to the hospital between January 2011 and July 2014. Isolates were initially identified by standard microbiological techniques in each hospital and then sent to a reference laboratory. The genus and species were confirmed using a species-specific multiplex PCR (48).

### Antibiotic and heavy metal susceptibility testing

Susceptibility testing to ciprofloxacin, clindamycin, cotrimoxazole, erythromycin, gentamicin, linezolid, rifampicin, tetracycline, trimethoprim, and vancomycin was performed using the agar diffusion method according to the 2024 CLSI guidelines (49). Ceftaroline susceptibility was determined by broth microdilution according to the 2024 CLSI guidelines (49). The MIC for Cu, As, Cd, and Hg was determined by the broth microdilution method using CuSO_4_ · 5H_2_O (Sigma-Aldrich), NaAsO_2_ (Sigma-Aldrich), CdSO_4_ (Sigma-Aldrich), and HgSO_4_ (Winkler), respectively. Briefly, 4–6 colonies were selected from a trypto-casein soy agar plate and disaggregated in 1.5 mL of sterile saline solution (NaCl 0.9%). The cellular suspension was adjusted to a 0.50 (±0.04) McFarland and diluted 1/200 in Mueller Hinton (MH) broth (BD) supplemented with increasing concentrations of each metal salt. The range of metal concentrations evaluated was as follows: 1.6–52 mg/L NaAsO_2_, 1.3–667 mg/L CdSO_4_, and 0.2–29.7 mg/L HgSO_4_. Assays were performed in round bottom 96-well plates (Costar) incubated for 18–20 h at 37°C. The lowest concentration of each compound resulting in undetectable visible growth was registered as the MIC value. All heavy metal MIC determinations were performed in duplicate with three biological replicates.

### Whole-genome sequencing

To study the genetic diversity of the ChC MRSA clone circulating in Latin America and explore the potential role of HMRGs in its dissemination, we performed whole-genome sequencing on our collection of 113 isolates. The genomic DNA of each isolate was obtained with the DNeasy Blood & Tissue Kit (Qiagen) from fresh overnight cultures after treatment with lysostaphin for 30 min at 37°C. DNA concentration was determined by the Qubit dsDNA HS Assay in the Qubit 2.0 fluorometer (Thermo Fisher Scientific). Genomic libraries were prepared using the NexteraXT DNA Sample Preparation Kit (Illumina). Sequencing was performed using HiSeq and MiSeq (Illumina) platforms, generating 150 or 300 base paired-end reads, respectively. The quality of raw reads was determined by FASTQC v0.11.9 and MultiQC v1.10.1 (50). Pairing was performed with Trimmomatic v0.39 after excluding reads with a quality score on a Phred scale below 30 (51). Finally, genomes were *de novo* assembled with SPAdes v3.13.0, and the quality of the assemblies was assessed with QUAST v5.0.2 (52, 53). To further obtain a closed genome and, thus, explore the genetic context of the HMRGs, the genomes of four representative strains were also subjected to LRS (MinION, Oxford Nanopore Technologies, Oxford, UK) using the SQK-LSK208 Kit following the manufacturer’s instructions. A consensus hybrid assembly using both Illumina and LRS reads was obtained with a custom Python pipeline (https://github.com/wshropshire/flye_hybrid_assembly_pipeline).

### Genetic characterization of MRSA isolates

*In silico* characterization of the sequence type (ST) and SCC*mec* cassettes was performed using MLST v2.19.0 and SCC*mec*Finder v12, respectively (54, 55). A personalized database was used to detect genes associated with heavy metal resistance with ABRicate (56). To evaluate the potential role of HMRGs in the divergence of ST5-SCC*mec*I MRSA, we performed a blast search of 22 HMRGs involved in the homeostatic processing of heavy metals: 13 out of these genes have been described as horizontally acquired (*arsC*, *arsR*, *cadA*, *cadC*, *cadD*, *cadX*, *copB*, *czrC*, *mco*, *merA*, *merB, merR*, and *merT*) and 9 as part of the core genome of *S. aureus* (*arsB*, *copA*, *czcD*, *czrA*, *czrB*, *fieF*, *isdA*, *isdB*, and *mntH*).

### Plasmid characterization and analyses

Plasmid characterization was performed with Plasmidfinder and PLSDB v2023_11_03_v2 (57, 58). The annotation and visualization of the pSCL4752 plasmid were performed with Prokka v1.0.0 13 and CGView Builder v1.0.0 in the Proksee web-based platform (https://proksee.ca) (59–61). The search for plasmids with high similarity to pSCL4752 was performed with the PLSDB v2023_11_03_v2 search tool (58). To evaluate the presence of pSCL4752 in our collection, we performed a blast search of the coding sequences included in the closed plasmid. Then, we extracted all the contigs that showed a partial match with the pSCL4752 coding sequences with MAUVE, and we performed an alignment with each sequence with the cPlot tool (62). All the alignments with an identity of over 90% were considered as pSCL4752 family plasmids.

### Phylogenomic analysis

Genome assemblies were annotated with Prokka v1.14, and the pangenome of the isolates was determined with Roary v3.13.0 (59, 63). A maximum likelihood (ML) phylogenetic tree using a core genome definition of 99% was performed with RAxML 8.2.12 with 100 bootstrap iterations using a general time reversible (GTR) substitution model with four gamma rate categories (64). Recombination events were assessed with Clonal Frame ML v1.12, using the core genome alignment and the ML tree (65). All phylogenomic trees were visualized with the interactive Tree Of Life (iTOL) v6 tool (66). We used the tool SNP-sites to identify single nucleotid polymorphisms (SNPs) present in the core genome, and a new core genome alignment including only the SNPs was generated (67). The substitution model to generate the molecular clock analysis was determined by IQ-TREE v1.6.12 (68). The evolutionary rates and dates of the most recent common ancestor were determined with BEAST v1.7.5 (69). The molecular clock was constructed using a GTR substitution model with empirical base frequencies, an exponential growth demographic model, and a strict clock model using flat priors between 10^−3^ and 10^−9^ substitutions/site/year. A Markov chain Monte Carlo analysis was performed with chains of 100,000,000 steps with sampling every 10,000 generations using a burn-in set at 10%. Models were evaluated with Tracer 1.7.1, and the consensus tree of maximum clade credibility was generated from three independent Bayesian inferences with LogCombiner 1.7.5 and TreeAnnotator 1.7.5 (70).

### Plasmid curing

The plasmid curing protocol consisted of consecutive 24 h passages of cultures growing with shaking at 44°C in tubes containing fresh MH broth (71). Each day, 10 µL from the overnight culture was spread with a sterile loop in mannitol salt agar and incubated at 37°C for 18 h. Single colonies were individually plated onto MH agar plates and incubated overnight at 37°C. PCR amplification of *merR*, the highly conserved response regulator of the Hg resistance gene cluster identified in the plasmid (not present in the chromosome), was used to screen the loss of the plasmid. Colonies whose amplification of the *merR* fragment was negative (strains with plasmid-cured) were further tested by broth microdilution to evaluate susceptibility to heavy metals as described above.

### Growth curves

Isolated colonies of each strain were grown overnight in MH broth at 37°C and then diluted in MH broth to an OD_600_ of 0.01 in flat transparent 96-well plates (Falcon). Plates were incubated by shaking at 37°C until 72 h in a Cytation 5 multi-reader (Biotek), where absorbance at OD_600_ was monitored every 15 min. The maximal point of growth (*K*) and doubling time (Tgen) for each experiment were calculated. All growth curves were performed with three biological replicates.

### Statistical analysis

Data are presented as means ± standard deviations from three experiments unless stated otherwise. The statistically significant association of categorical variables was determined by Fisher’s exact test. Statistical significance (*P* < 0.05) in MICs was assessed by the Mann-Whitney non-parametric test for the clinical isolates and with the Wilcoxon matched-pairs signed rank test for the isogenic strains. All statistical analyses were performed with the software GraphPad Prism 9.

## Data Availability

All genomes are publicly available in the U.S. National Center for Biotechnology Information Sequence Read Archive, in the BioProjects PRJNA291213 and PRJNA595928.

## SUPPLEMENTAL MATERIAL

Supplemental tables mbio.03669-24-s0001.pdf Tables S1 to S3.

ASM does not own the copyrights to Supplemental Material that may be linked to, or accessed through, an article. The authors have granted ASM a non-exclusive, world-wide license to publish the Supplemental Material files. Please contact the corresponding author directly for reuse.
