# Supplementary material for: Geographic divergence of methicillin-resistant Staphylococcus aureus ST5-SCCmecI in the aftermath of a major earthquake and tsunami: impact of a plasmid harboring heavy metal resistance genes
Source: mBio. 2025 Mar 19;16(4):e03669-24. doi: 10.1128/mbio.03669-24 (PMC11980568; doi:10.1128/mbio.03669-24)
Supplement: Supplemental tables — Tables S1 to S3. [file mbio.03669-24-s0001.pdf]

Supplementary table 1. Susceptibility of the isolates included in this study

							Susceptibility (MIC [µg/ml]/Interpretation)												
**Genome_ID**	**Isolate_ID**	**Country**	**City**	**ST**	**SCC*mec***	**Hospital**	**Oxacillin**	**Erythromycin**	**Clindamycin**	**Ciprofloxacin**	**Gentamicin**	**Tetracycline**	**Rifampicin**	**Vancomycin**	**Linezolide**	**Chloramphenicole**	**Minocycline**	**Trimethoprim-sulfamethoxazole**	**Ceftaroline**
UA872	4803	Argentina	Buenos Aires	5	I	UAHO2	>64/R	>64/R	>32/R	16/R	16/R	1/S	0.004/S	1/S	2/S	8/S	0.25/S	0.12/2.37/S	4/SDD
UB345	4830	Brazil	Sao Paulo	5	I	UBH01	>64/R	>64/R	>32/R	>32/R	32/R	2/S	0.004/S	1/S	2/S	32/R	0.5/S	0.12/2.37/S	0.25/S
UB354	4811	Brazil	Sao Paulo	5	I	UBH01	>64/R	>64/R	>32/R	>32/R	32/R	2/S	0.004/S	1/S	2/S	32/R	0.5/S	0.12/2.37/S	2/SDD
UB590	4718	Brazil	Sao Paulo	5	I	UBH02	>64/R	>64/R	>32/R	>32/R	0.12/S	1/S	0.004/S	1/S	2/S	64/R	0.06/S	0.06/1.18/S	2/SDD
UB591	4834	Brazil	Sao Paulo	5	I	UBH02	>64/R	64/R	>32/R	>32/R	>64/R	1/S	0.004/S	1/S	2/S	8/S	0.06/S	0.06/1.18/S	2/SDD
UC16	4753	Colombia	Bogotá	5	I	UCH01	>64/R	32/R	0.25/S	32/R	2/S	0.5/S	0.008/S	1/S	2/S	8/S	0.06/S	0.25/4.75/S	1/S
UCL1184	4799	Chile	Santiago	5	I	UCLH03	>64/R	>64/R	>32/R	>32/R	>64/R	0.5/S	0.008/S	1/S	2/S	8/S	0.25/S	0.06/1.18/S	2/SDD
UCL371	4847	Chile	Santiago	5	I	UCLH03	>64/R	>64/R	>32/R	16/R	0.5/S	0.5/S	0.004/S	1/S	2/S	8/S	0.5/S	0.06/1.18/S	2/SDD
UCL377	4729	Chile	Santiago	5	I	UCLH03	>64/R	>64/R	>32/R	32/R	>64/R	0.5/S	0.004/S	1/S	2/S	8/S	0.5/S	0.12/2.37/S	1/S
UCL380	4717	Chile	Santiago	5	I	UCLH03	>64/R	>64/R	>32/R	32/R	>64/R	0.5/S	0.25/S	1/S	2/S	64/R	0.5/S	0.12/2.37/S	2/SDD
UCL386	4754	Chile	Santiago	5	I	UCLH03	>64/R	>64/R	>32/R	32/R	>64/R	0.5/S	0.25/S	1/S	2/S	8/S	0.25/S	0.12/2.37/S	2/SDD
UCL398	4740	Chile	Santiago	5	I	UCLH03	>64/R	>64/R	>32/R	>32/R	>64/R	0.5/S	0.002/S	1/S	2/S	8/S	0.25/S	0.12/2.37/S	2/SDD
UCL401	4853	Chile	Santiago	105	II	UCLH03	64/R	>64/R	>32/R	>32/R	0.5/S	2/S	0.002/S	1/S	2/S	8/S	0.25/S	0.12/2.37/S	0.5/S
UCL406	4804	Chile	Santiago	5	I	UCLH03	>64/R	>64/R	>32/R	32/R	>64/R	1/S	0.002/S	1/S	2/S	8/S	0.25/S	0.12/2.37/S	2/SDD
UCL418	4760	Chile	Concepción	5	I	UCLH02	>64/R	>64/R	>32/R	>32/R	>64/R	2/S	0.002/S	1/S	2/S	8/S	0.25/S	0.12/2.37/S	2/SDD
UCL424	4730	Chile	Concepción	5	I	UCLH02	>64/R	>64/R	>32/R	>32/R	>64/R	4/S	0.002/S	1/S	2/S	8/S	0.25/S	0.12/2.37/S	2/SDD
UCL428	4839	Chile	Concepción	5	I	UCLH02	>64/R	>64/R	>32/R	16/R	>64/R	2/S	0.002/S	1/S	2/S	4/S	0.25/S	0.12/2.37/S	2/SDD
UCL436	4750	Chile	Concepción	5	I	UCLH02	>64/R	>64/R	>32/R	>32/R	>64/R	0.5/S	0.002/S	1/S	2/S	8/S	0.25/S	0.06/1.18/S	2/SDD
UCL440	4713	Chile	Concepción	5	I	UCLH02	>64/R	>64/R	>32/R	8/R	>64/R	0.25/S	0.002/S	1/S	2/S	8/S	0.25/S	0.06/1.18/S	2/SDD
UCL445	4755	Chile	Concepción	5	I	UCLH02	>64/R	>64/R	>32/R	32/R	>64/R	0.5/S	0.25/S	1/S	2/S	8/S	0.25/S	0.06/1.18/S	2/SDD
UCL450	4714	Chile	Concepción	5	I	UCLH02	>64/R	>64/R	>32/R	>32/R	>64/R	0.25/S	0.002/S	1/S	2/S	8/S	0.25/S	0.06/1.18/S	2/SDD
UCL454	4844	Chile	Concepción	5	I	UCLH02	>64/R	>64/R	>32/R	>32/R	>64/R	0.25/S	0.002/S	1/S	2/S	8/S	0.25/S	0.06/1.18/S	2/SDD
UCL720	4764	Chile	Santiago	5	I	UCLH01	>64/R	>64/R	>32/R	>32/R	>64/R	0.5/S	8/R	1/S	2/S	8/S	0.12/S	0.06/1.18/S	1/S
UP1027	4836	Perú	Lima	5	I	UPH02	>64/R	>64/R	>32/R	>32/R	>64/R	0.5/S	0.015/S	1/S	2/S	8/S	0.12/S	0.06/1.18/S	2/SDD
UP106	4835	Perú	Lima	5	I	UPH03	>64/R	>64/R	>32/R	16/R	32/R	16/R	0.015/S	1/S	2/S	8/S	0.5/S	0.25/4.75/S	4/SDD
UP1072	4848	Perú	Lima	5	I	UPH03	>64/R	>64/R	>32/R	>32/R	>64/R	0.5/S	0.004/S	1/S	2/S	64/R	0.25/S	0.12/2.37/S	4/SDD
UP703	n/a	Perú	Lima	5	I	UPH03	>64/R	>64/R	>32/R	32/R	>64/R	1/S	0.015/S	1/S	2/S	8/S	0.12/S	0.06/1.18/S	n/a
UP788	4837	Perú	Lima	5	I	UPH01	>64/R	>64/R	>32/R	32/R	>64/R	0.5/S	0.015/S	1/S	2/S	8/S	0.12/S	0.06/1.18/S	4/SDD
UP954	4747	Perú	Lima	5	I	UPH01	>64/R	>64/R	>32/R	32/R	>64/R	0.5/S	0.015/S	1/S	2/S	8/S	0.25/S	0.25/4.75/S	2/SDD
UV1155	4720	Venezuela	Caracas	5	I	UVH01	>64/R	>64/R	>32/R	32/R	16/R	0.5/S	0.015/S	1/S	2/S	8/S	0.12/S	0.06/1.18/S	2/SDD
UV165	4814	Venezuela	Caracas	5	IV	UVH01	32/R	32/R	0.06/S	0.5/S	1/S	16/R	0.008/S	1/S	2/S	8/S	0.12/S	0.5/9.57/S	0.5/S
UA796	4763	Argentina	Buenos Aires	5	I	UAHO1	>64/R	0.5/S	0.12/S	32/R	32/R	0.5/S	0.004/S	1/S	2/S	8/S	0.25/S	0.06/1.18/S	1/S
UCL356	4715	Chile	Santiago	5	I	UCLH03	>64/R	>64/R	>32/R	32/R	>64/R	0.12/S	0.004/S	1/S	2/S	8/S	0.5/S	0.06/1.18/S	2/SDD
UCL362	4722	Chile	Santiago	5	I	UCLH03	>64/R	>64/R	>32/R	32/R	>64/R	0.5/S	0.004/S	1/S	2/S	8/S	0.5/S	0.06/1.18/S	4/SDD
UCL381	4824	Chile	Santiago	5	I	UCLH03	>64/R	>64/R	>32/R	>32/R	>64/R	0.12/S	0.004/S	1/S	2/S	8/S	0.5/S	0.06/1.18/S	2/SDD
UCL383	4821	Chile	Santiago	5	I	UCLH03	>64/R	>64/R	>32/R	32/R	>64/R	0.5/S	0.002/S	1/S	2/S	8/S	0.25/S	0.12/2.37/S	2/SDD
UCL395	4823	Chile	Santiago	5	I	UCLH03	>64/R	>64/R	>32/R	>32/R	>64/R	2/S	0.002/S	1/S	2/S	8/S	0.25/S	0.12/2.37/S	2/SDD
UCL412	4719	Chile	Santiago	5	I	UCLH03	>64/R	>64/R	>32/R	>32/R	>64/R	4/S	0.002/S	1/S	2/S	8/S	0.25/S	0.12/2.37/S	2/SDD
UCL414	4734	Chile	Concepción	5	I	UCLH02	>64/R	>64/R	>32/R	>32/R	32/R	2/S	0.002/S	1/S	2/S	8/S	0.25/S	0.12/2.37/S	1/S
UCL420	4752	Chile	Concepción	5	I	UCLH02	>64/R	>64/R	>32/R	>32/R	>64/R	2/S	0.002/S	1/S	2/S	8/S	0.25/S	0.12/2.37/S	1/S
UCL430	4850	Chile	Concepción	5	I	UCLH02	>64/R	>64/R	>32/R	16/R	>64/R	0.25/S	0.002/S	1/S	2/S	8/S	0.25/S	0.06/1.18/S	2/SDD
UCL432	4941	Chile	Concepción	5	I	UCLH02	>64/R	>64/R	>32/R	16/R	>64/R	0.25/S	0.002/S	1/S	2/S	8/S	0.25/S	0.06/1.18/S	0.5/S
UCL438	4825	Chile	Concepción	5	I	UCLH02	>64/R	>64/R	>32/R	8/R	>64/R	0.25/S	0.002/S	1/S	2/S	8/S	0.25/S	0.06/1.18/S	2/SDD
UCL441	4817	Chile	Concepción	5	I	UCLH02	>64/R	>64/R	>32/R	8/R	>64/R	0.25/S	0.002/S	1/S	2/S	8/S	0.25/S	0.06/1.18/S	2/SDD
UCL443	4721	Chile	Concepción	5	I	UCLH02	>64/R	>64/R	>32/R	>32/R	>64/R	0.5/S	0.002/S	1/S	2/S	8/S	0.25/S	0.06/1.18/S	2/SDD
UCL446	4709	Chile	Concepción	5	I	UCLH02	>64/R	>64/R	>32/R	16/R	>64/R	0.25/S	0.002/S	1/S	2/S	8/S	0.25/S	0.06/1.18/S	2/SDD
UCL456	4802	Chile	Concepción	5	I	UCLH02	>64/R	>64/R	>32/R	>32/R	64/R	0.5/S	0.002/S	1/S	2/S	8/S	0.25/S	0.06/1.18/S	2/SDD
UCL461	4800	Chile	Concepción	5	I	UCLH02	>64/R	>64/R	>32/R	8/R	64/R	0.25/S	0.002/S	1/S	2/S	8/S	0.12/S	0.06/1.18/S	2/SDD
UP101	n/a	Perú	Lima	5	I	UPH02	>64/R	>64/R	>32/R	16/R	32/R	0.5/S	0.015/S	1/S	2/S	8/S	0.5/S	0.25/4.75/S	n/a
UP1150	4820	Perú	Lima	5	I	UPH01	>64/R	>64/R	>32/R	32/R	>64/R	1/S	0.015/S	1/S	2/S	8/S	0.12/S	0.06/1.18/S	2/SDD
UP1161	4838	Perú	Lima	5	I	UPH02	>64/R	>64/R	>32/R	>32/R	>64/R	1/S	0.015/S	1/S	2/S	8/S	0.12/S	0.06/1.18/S	2/SDD
UP118	4810	Perú	Lima	5	I	UPH03	>64/R	>64/R	>32/R	>32/R	>64/R	2/S	0.015/S	1/S	2/S	8/S	0.12/S	0.12/2.37/S	2/SDD
UP120	4706	Perú	Lima	5	I	UPH03	>64/R	>64/R	>32/R	16/R	>64/R	0.25/S	0.015/S	1/S	2/S	8/S	0.12/S	0.12/2.37/S	4/SDD
UP130	4744	Perú	Lima	5	I	UPH03	>64/R	>64/R	>32/R	16/R	>64/R	0.25/S	0.015/S	1/S	2/S	8/S	0.12/S	0.12/2.37/S	1/S
UP131	4842	Perú	Lima	5	I	UPH03	>64/R	>64/R	>32/R	32/R	>64/R	2/S	0.015/S	1/S	2/S	8/S	0.12/S	0.25/4.75/S	0.25/S
UP35	4845	Perú	Lima	5	I	UPH01	32/R	>64/R	>32/R	16/R	64/R	0.25/S	0.015/S	1/S	2/S	8/S	0.12/S	0.12/2.37/S	2/SDD
UP75	4812	Perú	Lima	5	I	UPH01	>64/R	>64/R	>32/R	>32/R	32/R	0.5/S	0.015/S	1/S	2/S	8/S	0.5/S	0.25/4.75/S	2/SDD
UP80	4819	Perú	Lima	5	I	UPH01	>64/R	>64/R	>32/R	16/R	32/R	0.25/S	0.008/S	1/S	2/S	8/S	0.5/S	0.25/4.75/S	2/SDD
UP83	4716	Perú	Lima	5	I	UPH01	>64/R	>64/R	>32/R	32/R	32/R	0.5/S	0.015/S	1/S	2/S	8/S	0.5/S	0.25/4.75/S	4/SDD

UP89	4711	Perú	Lima	5	I	UPH01	>64/R	>64/R	>32/R	16/R	32/R	0.25/S	0.015/S	1/S	2/S	8/S	0.5/S	0.12/2.37/S	2/SDD
UP91	4728	Perú	Lima	5	I	UPH02	>64/R	>64/R	>32/R	16/R	32/R	0.5/S	0.015/S	1/S	2/S	8/S	0.5/S	0.25/4.75/S	1/S
UP92	4881	Perú	Lima	5	I	UPH02	>64/R	>64/R	>32/R	16/R	32/R	0.5/S	0.015/S	1/S	2/S	8/S	0.5/S	0.25/4.75/S	1/S
UP93	4765	Perú	Lima	5	I	UPH02	>64/R	>64/R	>32/R	>32/R	32/R	0.5/S	0.015/S	1/S	2/S	8/S	1/S	0.12/2.37/S	1/S
UV156	4801	Venezuela	Caracas	5	I	UVH01	>64/R	>64/R	>32/R	>32/R	>64/R	1/S	0.008/S	1/S	2/S	8/S	0.12/S	1/19/S	2/SDD
UV157	4849	Venezuela	Caracas	5	I	UVH01	>64/R	>64/R	>32/R	>32/R	>64/R	1/S	0.008/S	1/S	2/S	8/S	0.12/S	1/19/S	2/SDD
UV160	4726	Venezuela	Caracas	5	IV	UVH01	8/R	32/R	0.06/S	0.5/S	1/S	64/R	0.008/S	1/S	2/S	8/S	0.12/S	0.5/9.57/S	0.5/S
UV164	n/a	Venezuela	Caracas	5	I	UVH01	>64/R	>64/R	>32/R	32/R	>64/R	1/S	0.008/S	1/S	2/S	8/S	0.12/S	1/19/S	n/a
UV166	4756	Venezuela	Caracas	5	I	UVH01	32/R	>64/R	>32/R	32/R	>64/R	1/S	0.008/S	1/S	2/S	8/S	0.12/S	0.5/9.57/S	1/S
UV171	4712	Venezuela	Caracas	5	I	UVH01	>64/R	>64/R	>32/R	>32/R	>64/R	0.5/S	0.008/S	1/S	2/S	8/S	0.12/S	0.5/9.57/S	2/SDD
UV177	4707	Venezuela	Caracas	5	I	UVH01	>64/R	>64/R	>32/R	>32/R	>64/R	1/S	0.008/S	1/S	2/S	8/S	0.12/S	0.5/9.57/S	2/SDD
UB544	4735	Brazil	Sao Paulo	5	I	UBH02	>64/R	>64/R	>32/R	>32/R	64/R	0.25/S	0.004/S	1/S	2/S	8/S	0.25/S	0.06/1.18/S	2/SDD
UC24	4767	Colombia	Bogotá	5	I	UCH02	>64/R	>64/R	>32/R	32/R	>64/R	0.5/S	0.008/S	1/S	2/S	8/S	0.25/S	0.25/4.75/S	0.5/S
UC45	4796	Colombia	Bogotá	5	I	UCH01	>64/R	>64/R	>32/R	32/R	>64/R	0.5/S	0.015/S	1/S	2/S	8/S	0.12/S	0.5/9.57/S	2/SDD
UCL1179	4742	Chile	Santiago	5	I	UCLH03	>64/R	>64/R	>32/R	>32/R	>64/R	0.5/S	0.008/S	1/S	2/S	8/S	0.12/S	0.06/1.18/S	2/SDD
UCL1181	4833	Chile	Santiago	5	I	UCLH03	>64/R	>64/R	>32/R	>32/R	>64/R	0.5/S	0.008/S	1/S	2/S	8/S	0.25/S	0.06/1.18/S	2/SDD
UCL1182	4736	Chile	Santiago	5	I	UCLH03	>64/R	>64/R	>32/R	>32/R	>64/R	0.5/S	0.008/S	1/S	2/S	8/S	0.25/S	0.06/1.18/S	2/SDD
UCL1185	4808	Chile	Santiago	5	I	UCLH03	>64/R	>64/R	>32/R	>32/R	>64/R	0.5/S	0.008/S	1/S	2/S	8/S	0.25/S	0.06/1.18/S	2/SDD
UCL379	4710	Chile	Santiago	5	I	UCLH03	>64/R	>64/R	>32/R	32/R	>64/R	0.5/S	0.004/S	1/S	2/S	8/S	0.5/S	0.12/2.37/S	4/SDD
UCL387	4816	Chile	Santiago	5	I	UCLH03	>64/R	>64/R	>32/R	32/R	>64/R	1/S	0.002/S	1/S	2/S	8/S	0.25/S	0.12/2.37/S	2/SDD
UCL392	4798	Chile	Santiago	5	I	UCLH03	>64/R	>64/R	>32/R	>32/R	64/R	0.5/S	0.002/S	1/S	2/S	8/S	0.25/S	0.12/2.37/S	2/SDD
UCL394	4741	Chile	Santiago	5	I	UCLH03	>64/R	>64/R	>32/R	32/R	>64/R	2/S	0.002/S	1/S	2/S	8/S	0.25/S	0.12/2.37/S	2/SDD
UCL396	4759	Chile	Santiago	5	I	UCLH03	>64/R	>64/R	>32/R	>32/R	>64/R	2/S	0.002/S	1/S	2/S	8/S	0.25/S	0.12/2.37/S	2/SDD
UCL397	n/a	Chile	Santiago	5	I	UCLH03	>64/R	>64/R	>32/R	>32/R	>64/R	2/S	0.002/S	1/S	2/S	8/S	0.25/S	0.12/2.37/S	n/a
UCL400	4758	Chile	Santiago	5	I	UCLH03	>64/R	>64/R	>32/R	>32/R	32/R	2/S	0.002/S	1/S	2/S	8/S	0.25/S	0.12/2.37/S	2/SDD
UCL410	4746	Chile	Santiago	5	I	UCLH03	>64/R	>64/R	>32/R	>32/R	>64/R	2/S	0.002/S	1/S	2/S	8/S	0.25/S	0.12/2.37/S	2/SDD
UCL411	4766	Chile	Santiago	5	I	UCLH03	>64/R	>64/R	>32/R	>32/R	>64/R	2/S	0.002/S	1/S	2/S	8/S	0.25/S	0.12/2.37/S	1/S
UCL413	4843	Chile	Concepción	5	I	UCLH02	>64/R	>64/R	>32/R	>32/R	>64/R	2/S	0.002/S	1/S	2/S	8/S	0.25/S	0.12/2.37/S	2/SDD
UCL417	4762	Chile	Concepción	5	I	UCLH02	>64/R	>64/R	>32/R	>32/R	32/R	4/S	0.002/S	1/S	2/S	8/S	0.25/S	0.12/2.37/S	1/S
UCL449	4813	Chile	Concepción	5	I	UCLH02	>64/R	>64/R	>32/R	8/R	64/R	0.5/S	0.002/S	1/S	2/S	8/S	0.25/S	0.06/1.18/S	2/SDD
UCL451	4732	Chile	Concepción	5	I	UCLH02	>64/R	>64/R	>32/R	16/R	>64/R	0.25/S	0.002/S	1/S	2/S	8/S	0.25/S	0.06/1.18/S	1/S
UCL452	4737	Chile	Concepción	5	I	UCLH02	>64/R	>64/R	>32/R	>32/R	>64/R	0.25/S	0.002/S	1/S	2/S	8/S	0.25/S	0.06/1.18/S	2/SDD
UCL453	4761	Chile	Concepción	5	I	UCLH02	>64/R	>64/R	>32/R	>32/R	>64/R	0.25/S	0.002/S	1/S	2/S	8/S	0.25/S	0.06/1.18/S	2/SDD
UCL462	4852	Chile	Concepción	5	I	UCLH02	>64/R	>64/R	>32/R	8/R	64/R	0.5/S	0.002/S	1/S	2/S	8/S	0.12/S	0.06/1.18/S	1/S
UP100	4851	Perú	Lima	5	I	UPH02	>64/R	>64/R	>32/R	16/R	32/R	0.5/S	0.015/S	1/S	2/S	8/S	0.5/S	0.25/4.75/S	1/S
UP102	4727	Perú	Lima	5	I	UPH02	>64/R	>64/R	>32/R	>32/R	32/R	0.5/S	0.015/S	1/S	2/S	8/S	0.5/S	0.12/2.37/S	2/SDD
UP103	4832	Perú	Lima	5	I	UPH03	>64/R	>64/R	>32/R	>32/R	32/R	0.5/S	0.015/S	1/S	2/S	8/S	0.5/S	0.25/4.75/S	2/SDD
UP112	4733	Perú	Lima	5	I	UPH03	>64/R	>64/R	>32/R	16/R	32/R	0.25/S	0.015/S	1/S	2/S	8/S	1/S	0.25/4.75/S	2/SDD
UP1140	4731	Perú	Lima	5	I	UPH01	>64/R	>64/R	>32/R	>32/R	>64/R	1/S	0.015/S	1/S	2/S	8/S	0.12/S	0.12/2.37/S	1/S
UP115	4840	Perú	Lima	5	I	UPH03	>64/R	>64/R	>32/R	16/R	32/R	1/S	0.03/S	1/S	2/S	8/S	1/S	0.25/4.75/S	2/SDD
UP1152	n/a	Perú	Lima	5	I	UPH02	>64/R	>64/R	>32/R	>32/R	>64/R	1/S	0.015/S	1/S	2/S	8/S	0.12/S	0.12/2.37/S	n/a
UP117	4854	Perú	Lima	5	I	UPH03	>64/R	>64/R	>32/R	32/R	>64/R	0.25/S	0.015/S	1/S	2/S	8/S	0.12/S	0.12/2.37/S	0.25/S
UP123	4708	Perú	Lima	5	I	UPH03	>64/R	>64/R	>32/R	32/R	>64/R	0.25/S	0.015/S	1/S	2/S	8/S	0.12/S	0.12/2.37/S	2/SDD
UP125	4841	Perú	Lima	5	I	UPH03	>64/R	>64/R	>32/R	16/R	>64/R	0.25/S	0.015/S	1/S	2/S	8/S	0.12/S	0.12/2.37/S	2/SDD
UP129	4723	Perú	Lima	5	I	UPH03	>64/R	>64/R	>32/R	16/R	>64/R	0.5/S	0.015/S	1/S	2/S	8/S	0.12/S	0.12/2.37/S	1/S
UP133	4748	Perú	Lima	5	I	UPH03	>64/R	>64/R	>32/R	16/R	>64/R	0.5/S	0.015/S	1/S	2/S	8/S	0.12/S	0.12/2.37/S	2/SDD
UP71	4724	Perú	Lima	5	I	UPH01	>64/R	>64/R	>32/R	>32/R	32/R	64/R	0.015/S	1/S	2/S	8/S	0.5/S	0.12/2.37/S	1/S
UP73	4743	Perú	Lima	5	I	UPH01	>64/R	>64/R	>32/R	32/R	32/R	1/S	0.008/S	1/S	2/S	8/S	1/S	0.25/4.75/S	2/SDD
UP90	4822	Perú	Lima	5	I	UPH02	>64/R	>64/R	>32/R	16/R	32/R	0.25/S	0.015/S	1/S	2/S	8/S	0.5/S	0.12/2.37/S	4/SDD
UP97	4805	Perú	Lima	5	I	UPH02	>64/R	>64/R	>32/R	16/R	32/R	0.5/S	0.015/S	1/S	2/S	8/S	0.5/S	0.25/4.75/S	4/SDD
UV1156	4725	Venezuela	Caracas	5	I	UVH01	>64/R	>64/R	>32/R	32/R	>64/R	1/S	0.015/S	1/S	2/S	8/S	0.12/S	0.003/0.6/S	2/SDD
UV162	4815	Venezuela	Caracas	5	I	UVH01	32/R	>64/R	>32/R	32/R	32/R	0.5/S	0.004/S	1/S	2/S	8/S	0.12/S	1/19/S	2/SDD
UV168	4797	Venezuela	Caracas	5	I	UVH01	>64/R	>64/R	>32/R	32/R	>64/R	1/S	0.008/S	1/S	2/S	8/S	0.12/S	1/19/S	0.25/S
UV175	4818	Venezuela	Caracas	5	IV	UVH01	>64/R	64/R	0.12/S	0.5/S	2/S	32/R	0.008/S	1/S	2/S	8/S	0.12/S	0.5/9.57/S	1/S

Supplementary table 2. Antimicrobial resistance genes present in the 113 MRSA genomes (1: presence, 0: absence)

	mecA	*blaZ*	*tet(38)*	*fosB*	*mepA*	*lmrS*	*ant(9)-Ia*	*erm(A)*	*aph(3')-IIIa*	*sat4*	*ant(6)-Ia*	*aac(6')-Ie/aph(2'')-Ia*	*bleO*	*aadD1*	*qacC*	*erm(C)*	*msr(A)*	*tet(K)*	*lnu(A)*	*mph(C)*	*catA*	*mupA*	*blaI*	*blaR1*	*mecI*	*mecR1*
UA796	1	1	1	1	1	1	0	0	1	1	1	1	0	0	0	0	0	0	0	0	0	0	1	1	0	1
UA872	1	1	1	1	1	1	1	1	1	1	1	1	0	0	0	0	0	0	0	0	0	0	1	0	0	1
UB345	1	0	1	1	1	1	1	1	1	1	1	1	0	0	0	0	0	0	0	0	0	0	0	0	0	1
UB354	1	0	1	1	1	1	1	1	1	1	1	1	0	0	0	0	0	0	0	0	0	0	0	0	0	1
UB544	1	1	1	1	1	1	1	1	1	1	1	1	0	0	0	0	0	0	0	0	0	0	1	1	0	1
UB590	1	0	1	1	1	1	1	1	1	1	1	1	0	0	0	0	0	0	0	0	0	0	0	0	0	1
UB591	1	0	1	1	1	1	0	0	1	1	1	0	0	0	0	0	0	0	0	0	0	0	0	0	0	1
UC16	1	1	1	1	1	1	0	0	1	1	1	0	0	0	0	0	0	0	0	0	0	0	1	1	0	1
UC24	1	1	1	1	1	1	1	1	1	1	1	1	0	0	0	0	0	0	0	0	0	0	1	1	0	1
UC45	1	1	1	1	1	1	1	1	1	1	1	1	0	0	0	0	0	0	0	0	0	0	1	1	0	1
UCL1179	1	1	1	1	1	1	1	1	0	0	0	1	1	0	0	0	0	0	0	0	0	0	1	1	0	1
UCL1181	1	1	1	1	1	1	1	1	1	1	1	1	0	0	0	0	0	0	0	0	0	0	1	1	0	1
UCL1182	1	1	1	1	1	1	1	1	1	1	1	1	1	0	0	0	0	0	0	0	0	0	1	1	0	1
UCL1184	1	0	1	1	1	1	1	1	1	1	1	1	0	0	0	0	0	0	0	0	0	0	0	0	0	1
UCL1185	1	1	1	1	1	1	1	1	1	1	1	1	0	0	0	0	0	0	0	0	0	0	1	1	0	1
UCL356	1	1	1	1	1	1	1	1	1	1	1	1	0	0	0	0	0	0	0	0	0	0	1	1	0	1
UCL362	1	1	1	1	1	1	1	1	0	0	0	1	0	0	0	0	0	0	0	0	0	0	1	1	0	1
UCL371	1	1	1	1	1	1	1	1	1	1	1	0	0	0	0	0	0	0	0	0	0	0	1	1	0	1
UCL377	1	1	1	1	1	1	1	1	0	0	0	0	0	0	0	0	0	0	0	0	0	0	1	1	0	1
UCL379	1	1	1	1	1	1	1	1	0	0	0	1	0	0	0	0	0	0	0	0	0	0	1	1	0	1
UCL380	1	1	1	1	1	1	1	1	1	1	1	1	1	1	0	0	0	0	0	0	1	0	1	1	0	1
UCL381	1	1	1	1	1	1	1	1	1	1	1	1	1	0	0	0	0	0	0	0	0	0	1	1	0	1
UCL383	1	1	1	1	1	1	1	1	1	1	1	0	0	0	0	0	0	0	0	0	0	0	1	1	0	1
UCL386	1	1	1	1	1	1	1	1	1	1	1	1	0	0	0	0	0	0	0	0	0	0	1	1	0	1
UCL387	1	1	1	1	1	1	1	1	0	0	0	1	0	0	0	0	0	0	0	0	0	0	1	1	0	1
UCL392	1	1	1	1	1	1	1	1	0	0	0	1	0	0	0	0	0	0	0	0	0	0	1	1	0	1
UCL394	1	1	1	1	1	1	1	1	0	0	0	1	0	0	0	0	0	0	0	0	0	0	1	1	0	1
UCL395	1	1	1	1	1	1	1	1	0	0	0	1	0	0	0	0	0	0	0	0	0	0	1	1	0	1
UCL396	1	1	1	1	1	1	1	1	0	0	0	1	0	0	0	1	0	0	0	0	0	0	1	1	0	1
UCL397	1	1	1	1	1	1	1	1	1	1	1	1	0	0	1	0	0	0	0	0	0	0	1	1	0	1
UCL398	1	1	1	1	1	1	1	1	0	0	0	1	0	0	0	0	0	0	0	0	0	0	1	1	0	1
UCL400	1	0	1	1	1	1	1	1	1	1	1	1	0	0	0	0	0	0	0	0	0	0	0	0	0	1
UCL401	1	1	1	1	1	1	1	1	0	0	0	0	1	1	0	0	0	0	0	0	0	0	1	1	1	1
UCL406	1	1	1	1	1	1	1	1	0	0	0	1	0	0	0	0	0	0	0	0	0	0	1	1	0	1
UCL410	1	1	1	1	1	1	1	1	0	0	0	0	0	0	0	0	0	0	0	0	0	0	1	1	0	1
UCL411	1	1	1	1	1	1	1	1	0	0	0	0	0	0	0	0	0	0	0	0	0	0	1	1	0	1
UCL412	1	1	1	1	1	1	1	1	0	0	0	1	0	0	0	0	0	0	0	0	0	0	1	1	0	1
UCL413	1	1	1	1	1	1	1	1	1	1	1	1	0	0	0	0	0	0	0	0	0	0	1	1	0	1
UCL414	1	1	1	1	1	1	1	1	1	1	1	1	0	0	0	0	0	0	0	0	0	0	1	0	0	1
UCL417	1	1	1	1	1	1	1	1	1	1	1	1	0	0	0	0	0	0	0	0	0	0	1	1	0	1
UCL418	1	1	1	1	1	1	1	1	1	1	1	1	0	0	0	0	0	0	0	0	0	0	1	1	0	1
UCL420	1	1	1	1	1	1	1	1	1	1	1	1	0	0	0	0	0	0	0	0	0	0	1	1	0	1
UCL424	1	1	1	1	1	1	1	1	1	1	1	1	0	0	0	0	0	0	0	0	0	0	1	1	0	1
UCL428	1	0	1	1	1	1	1	1	1	1	1	1	0	0	0	0	0	0	1	0	0	0	0	0	0	1
UCL430	1	1	1	1	1	1	1	1	1	1	1	0	0	0	0	0	0	0	0	0	0	0	1	1	0	1
UCL432	1	1	1	1	1	1	1	1	1	1	1	1	0	0	0	0	0	0	0	0	0	0	1	1	0	1
UCL436	1	1	1	1	1	1	1	1	1	1	1	0	0	0	0	0	0	0	0	0	0	0	0	1	0	1
UCL438	1	1	1	1	1	1	1	1	1	1	1	1	0	0	0	0	0	0	0	0	0	0	1	1	0	1
UCL440	1	1	1	1	1	1	1	1	1	1	1	1	0	0	0	0	0	0	0	0	0	0	1	1	0	1
UCL441	1	1	1	1	1	1	1	1	1	1	1	1	0	0	0	0	0	0	0	0	0	0	1	1	0	1
UCL443	1	0	1	1	1	1	1	1	1	1	1	1	0	0	0	0	0	0	0	0	0	0	1	0	0	1
UCL445	1	1	1	1	1	1	1	1	1	1	1	1	0	0	0	0	0	0	0	0	0	0	1	1	0	1
UCL446	1	1	1	1	1	1	1	1	1	1	1	1	0	0	0	0	0	0	0	0	0	0	1	1	0	1
UCL449	1	1	1	1	1	1	1	1	1	1	1	1	0	0	0	0	0	0	0	0	0	0	1	1	0	1

UCL450	1	1	1	1	1	1	1	1	1	1	1	1	0	0	0	0	0	0	0	0	0	0	1	1	0	1
UCL451	1	1	1	1	1	1	1	1	1	1	1	1	1	0	0	0	0	0	0	0	0	0	1	1	0	1
UCL452	1	1	1	1	1	1	1	1	1	1	1	1	0	0	0	0	0	0	0	0	0	0	1	1	0	1
UCL453	1	1	1	1	1	1	1	1	1	1	1	0	0	0	0	0	0	0	0	0	0	0	1	1	0	1
UCL454	1	1	1	1	1	1	1	1	1	1	1	1	0	0	0	0	0	0	0	0	0	0	1	1	0	1
UCL456	1	0	1	1	1	1	1	1	1	1	1	1	0	0	0	0	0	0	0	0	0	0	0	0	0	1
UCL461	1	1	1	1	1	1	1	1	1	1	1	1	0	0	0	0	0	0	0	0	0	0	1	1	0	1
UCL462	1	1	1	1	1	1	1	1	1	1	1	1	0	0	0	0	0	0	0	0	0	0	1	1	0	1
UCL720	1	1	1	1	1	1	1	1	1	1	1	1	0	1	0	0	0	0	0	0	0	0	1	1	0	1
UP100	1	1	1	1	1	1	0	0	1	1	1	1	0	1	0	1	0	0	0	0	0	0	1	1	0	1
UP101	1	1	1	1	1	1	0	0	1	1	1	1	0	0	0	1	0	0	0	0	0	0	1	1	0	1
UP102	1	1	1	1	1	1	1	1	1	1	1	0	0	0	0	0	0	0	0	0	0	0	1	1	0	1
UP1027	1	0	1	1	1	1	1	1	1	1	1	1	0	0	0	0	0	0	0	0	0	0	0	0	0	1
UP103	1	1	1	1	1	1	1	1	1	1	1	1	0	0	0	0	0	0	0	0	0	0	1	1	0	1
UP106	1	1	1	1	1	1	1	1	1	1	1	1	0	0	0	0	0	0	0	0	0	0	1	1	0	1
UP1072	1	1	1	1	1	1	1	1	1	1	1	1	0	0	0	1	0	0	0	0	1	0	1	1	0	1
UP112	1	1	1	1	1	1	1	1	1	1	1	1	1	0	0	0	0	0	0	0	0	0	1	1	0	1
UP1140	1	1	1	1	1	1	1	1	1	1	1	1	0	0	0	0	0	0	0	0	0	0	1	1	0	1
UP115	1	1	1	1	1	1	1	1	1	1	1	1	0	0	0	0	0	0	0	0	0	0	1	1	0	1
UP1150	1	1	1	1	1	1	1	1	1	1	1	1	0	0	0	0	0	0	0	0	0	0	1	1	0	1
UP1152	1	1	1	1	1	1	1	1	1	1	1	1	0	1	0	0	0	0	0	0	0	0	1	1	0	1
UP1161	1	0	1	1	1	1	1	1	1	1	1	1	0	0	1	0	0	0	0	0	0	0	0	0	0	1
UP117	1	1	1	1	1	1	1	1	1	1	1	1	0	0	0	0	0	0	0	0	0	0	1	1	0	1
UP118	1	1	1	1	1	1	1	1	1	1	1	1	0	0	0	0	0	0	0	0	0	0	1	1	0	1
UP120	1	1	1	1	1	1	1	1	1	1	1	1	0	0	0	0	0	0	0	0	0	0	1	0	0	1
UP123	1	1	1	1	1	1	1	1	1	1	1	1	0	0	0	0	0	0	0	0	0	0	1	1	0	1
UP125	1	1	1	1	1	1	1	1	1	1	1	0	0	0	0	0	0	0	0	0	0	0	1	1	0	1
UP129	1	0	1	1	1	1	1	1	1	1	1	1	0	0	0	0	0	0	0	0	0	0	0	0	0	1
UP130	1	1	1	1	1	1	1	1	1	1	1	1	0	0	0	0	0	0	0	0	0	0	1	1	0	1
UP131	1	1	1	1	1	1	1	1	1	1	1	1	0	0	0	0	0	0	0	0	0	0	1	1	0	1
UP133	1	1	1	1	1	1	1	1	1	1	1	1	0	0	0	0	0	0	0	0	0	0	1	1	0	1
UP35	1	0	1	1	1	1	1	1	0	0	0	1	0	0	0	0	0	0	0	0	0	0	0	0	0	1
UP703	1	1	1	1	1	1	1	1	0	0	0	1	0	0	0	0	0	0	0	0	0	0	1	1	0	1
UP71	1	1	1	1	1	1	1	1	1	1	1	0	0	0	0	0	0	0	0	0	0	0	1	1	0	1
UP73	1	0	1	1	1	1	1	1	1	1	1	0	0	0	0	0	0	0	0	0	0	0	0	0	0	1
UP75	1	1	1	1	1	1	1	1	1	1	1	1	0	0	0	0	0	0	0	0	0	0	1	1	0	1
UP788	1	1	1	1	1	1	1	1	1	1	1	1	0	0	0	0	0	0	0	0	0	0	1	1	0	1
UP80	1	1	1	1	1	1	1	1	1	1	1	0	0	0	0	0	0	0	0	0	0	0	1	1	0	1
UP83	1	1	1	1	1	1	1	1	1	1	1	0	0	0	0	0	0	0	0	0	0	0	1	1	0	1
UP89	1	1	1	1	1	1	1	1	1	1	1	1	0	1	0	0	0	0	0	0	0	1	1	1	0	1
UP90	1	0	1	1	1	1	1	1	1	1	1	0	0	0	0	0	0	0	0	0	0	0	0	0	0	1
UP91	1	1	1	1	1	1	1	1	1	1	1	1	0	0	0	0	0	0	0	0	0	0	1	1	0	1
UP92	1	1	1	1	1	1	1	1	1	1	1	1	0	0	0	0	0	0	0	0	0	0	1	1	0	1
UP93	1	0	1	1	1	1	1	1	1	1	1	1	0	0	0	0	0	0	0	0	0	0	0	0	0	1
UP954	1	0	1	1	1	1	1	1	1	1	1	1	0	0	0	0	0	0	0	0	0	0	0	0	0	1
UP97	1	1	1	1	1	1	1	1	1	1	1	1	0	0	0	0	0	0	0	0	0	0	1	1	0	1
UV1155	1	1	1	1	1	1	1	1	1	1	1	1	0	0	0	0	0	0	0	0	0	0	1	1	0	1
UV1156	1	1	1	1	1	1	1	1	0	0	0	1	0	0	1	0	0	0	1	0	0	0	1	1	0	1
UV156	1	1	1	1	1	1	1	1	1	1	1	1	0	0	0	0	0	0	0	0	0	0	1	1	0	1
UV157	1	1	1	1	1	1	1	1	1	1	1	1	0	0	0	0	0	0	0	0	0	0	1	1	0	1
UV160	1	1	1	1	1	1	0	0	0	0	0	0	0	0	0	0	1	1	1	1	0	0	1	1	0	1
UV162	1	1	1	1	1	1	1	1	1	1	1	1	0	0	0	0	0	0	0	0	0	0	1	1	0	1
UV164	1	1	1	1	1	1	1	1	1	1	1	1	0	0	0	0	0	0	0	0	0	0	1	1	0	1
UV165	1	1	1	1	1	1	0	0	1	1	1	0	0	0	1	0	1	1	0	1	0	0	1	1	0	1
UV166	1	1	1	1	1	1	1	1	1	1	1	1	0	0	0	0	0	0	0	0	0	0	1	1	0	1
UV168	1	1	1	1	1	1	1	1	0	0	0	1	0	0	0	0	0	0	0	0	0	0	1	1	0	1

UV171	1	1	1	1	1	1	1	1	1	1	1	1	0	0	0	0	0	0	0	0	0	0	1	1	0	1
UV175	1	0	1	1	1	1	0	0	1	1	1	0	0	0	1	0	1	1	0	1	0	0	0	0	0	1
UV177	1	1	1	1	1	1	1	1	1	1	1	0	0	0	0	0	0	0	0	0	0	0	1	1	0	1

Supplementary table 3. Chromosome and plasmid size for the 4 ONT sequences.

GenomeID	City - Country	ChC-Clade	Chromosome size	Plasmid size
**UCL420**	Concepción- Chile	ChC-IIIa	3,018,843 bp	33,660 bp
**UC45**	Bogotá - Colombia	ChC-IIIb	2,928,124 bp	37,273 bp
**UCL417**	Concepción- Chile	ChC-IIIa	2,938,044 bp	37,108 bp
**UP788**	Lima - Perú	ChC-IIIc	2,981,209 bp	37,277 bp
